# Defining a novel *k*-nearest neighbours approach to assess the applicability domain of a QSAR model for reliable predictions

**DOI:** 10.1186/1758-2946-5-27

**Published:** 2013-05-30

**Authors:** Faizan Sahigara, Davide Ballabio, Roberto Todeschini, Viviana Consonni

**Affiliations:** 1 Milano Chemometrics and QSAR Research Group, Department of Earth and Environmental Sciences, University of Milano-Bicocca, P.za della Scienza 1, Milano 20126, Italy

**Keywords:** QSAR, Applicability domain, kNN, Nearest neighbour, Model validation

## Abstract

**Background:**

With the growing popularity of using QSAR predictions towards regulatory purposes, such predictive models are now required to be strictly validated, an essential feature of which is to have the model’s Applicability Domain (AD) defined clearly. Although in recent years several different approaches have been proposed to address this goal, no optimal approach to define the model’s AD has yet been recognized.

**Results:**

This study proposes a novel descriptor-based AD method which accounts for the data distribution and exploits *k*-Nearest Neighbours (kNN) principle to derive a heuristic decision rule. The proposed method is a three-stage procedure to address several key aspects relevant in judging the reliability of QSAR predictions. Inspired from the adaptive kernel method for probability density function estimation, the first stage of the approach defines a pattern of thresholds corresponding to the various training samples and these thresholds are later used to derive the decision rule. Criterion deciding if a given test sample will be retained within the AD is defined in the second stage of the approach. Finally, the last stage tries reflecting upon the reliability in derived results taking model statistics and prediction error into account.

**Conclusions:**

The proposed approach addressed a novel strategy that integrated the kNN principle to define the AD of QSAR models. Relevant features that characterize the proposed AD approach include: a) adaptability to local density of samples, useful when the underlying multivariate distribution is asymmetric, with wide regions of low data density; b) unlike several kernel density estimators (KDE), effectiveness also in high-dimensional spaces; c) low sensitivity to the smoothing parameter *k*; and d) versatility to implement various distances measures. The results derived on a case study provided a clear understanding of how the approach works and defines the model’s AD for reliable predictions.

## Background

The popularity of QSARs has seen a growth from time to time and was complemented by the availability of more sophisticated and efficient model development techniques. This fact was further supported by the consideration of QSAR predictions for regulatory purposes. To deal with risk assessment of chemicals for their safe use, a new European legislation – REACH (Registration, Evaluation, Authorization and restriction of Chemicals) was approved in the recent years [1]. To reduce animal testing and replacing them by cost effective methods, this law encourages the use of QSARs as a possible alternative when enough experimental data is not available, provided that the model was strictly validated for its regulatory consideration [2].

There are several aspects that must be taken into account before considering a QSAR model reliable enough. In other words, the validity of a model has to be evaluated. Existing literature has often emphasized upon validating the QSAR models to reflect their robustness and predictive ability. In 2004, following five OECD principles for model validation were adopted to validate a QSAR model for its regulatory consideration: a) a defined endpoint; b) an unambiguous algorithm; c) a defined domain of applicability d) appropriate measures for goodness-of-fit, robustness and predictivity and e) mechanistic interpretation, if possible [3].

Applicability domain (AD) of a QSAR model defines the model’s limitation in its structural domain and response space. In other words, this principle for model validation restricts the applicability of a model to reliably predict those test samples that are structurally similar to the training samples used to build that model [4-6]. Several approaches were proposed in the past years to define the AD of QSAR models. These approaches mainly differed in the algorithm used to characterise the AD within the descriptor space, where the model can predict reliably [7,8]. For instance, some classical approaches suggested defining the domain of applicability by a) considering the range of descriptors values; b) enclosing the training space in a convex hull; c) calculating the distance of a query compound from a defined point within the model’s descriptor space and d) estimating the Probability Density Function for the given data. All these approaches were associated with their own advantages and limitations [2,7-10]. From time to time, several approaches were proposed that were aimed to be more efficient or were thought to overcome several limitations of existing approaches.

This article proposes a new heuristic approach towards defining the AD of QSAR models. The basis of this novel strategy is inspired from the *k*-Nearest Neighbours (kNN) approach and adaptive kernel methods for probability density estimation (kernel density estimators, KDE) [11]. Due to its simplicity and easy implementation, kNN had been a preferred choice for several proposed QSAR studies [6,12-18].

In the classical kNN approach for AD evaluation [6,18], average distances of all the training samples from their *k* nearest neighbours are calculated and used to define a unique threshold to decide if a test sample is inside or outside the model’s AD (for example, 95th percentile). Moreover, in the framework of the probability density function estimation, the nearest neighbour method provides density estimates depending on the Euclidean distance to the *k*-th nearest data point [19]. Following the same concept, the proposed method tries to integrate the kNN principle with the salient features of adaptive kernel methods [11], which define local bandwidth factors corresponding to the training data points and use them to build the density estimate at a given point.

The novelty of the kNN based AD approach proposed in this article lies in the overall strategy that is properly executed in a three-stage procedure to encapsulate and reflect upon several significant aspects towards model validation. Moreover, some features common to most of the AD approaches were dealt differently with this approach; for instance, rather than defining a general threshold as in all the distance-based approaches, each training sample in this approach was associated with its individual threshold; in order to find an optimal smoothing parameter *k*, this approach performed a *k*-optimization procedure based on Monte Carlo validation; additionally, model’s statistical parameters and other relevant aspects were dealt simultaneously to reflect upon the reliability in the derived results.

To better understand the strategy behind this approach, it was implemented on a dataset from the literature. The dataset was chosen from the CAESAR project to predict the bioconcentration factor (BCF) [20,21].The derived results were discussed in comparison with those derived from other literature AD approaches.

## Methods

### *k*-Nearest Neighbours principle from AD perspective

The kNN principle basically reflects upon the structural similarity of a test sample to the training samples used to build that model. In theory, the distance of a query sample is considered from its *k* closest data points in the chemical space. Lower distance values correspond to a higher similarity, while the increasing distances signify higher levels of structural mismatch. The *k* value plays a significant role in defining how constraint the approach will be and thus, it can be referred to as the smoothing parameter.

A stepwise execution of the following three stages characterises the workflow of this approach:

1) defining thresholds for training samples

2) evaluating AD for new/test samples

3) optimizing the smoothing parameter *k*

**Figure 1:**
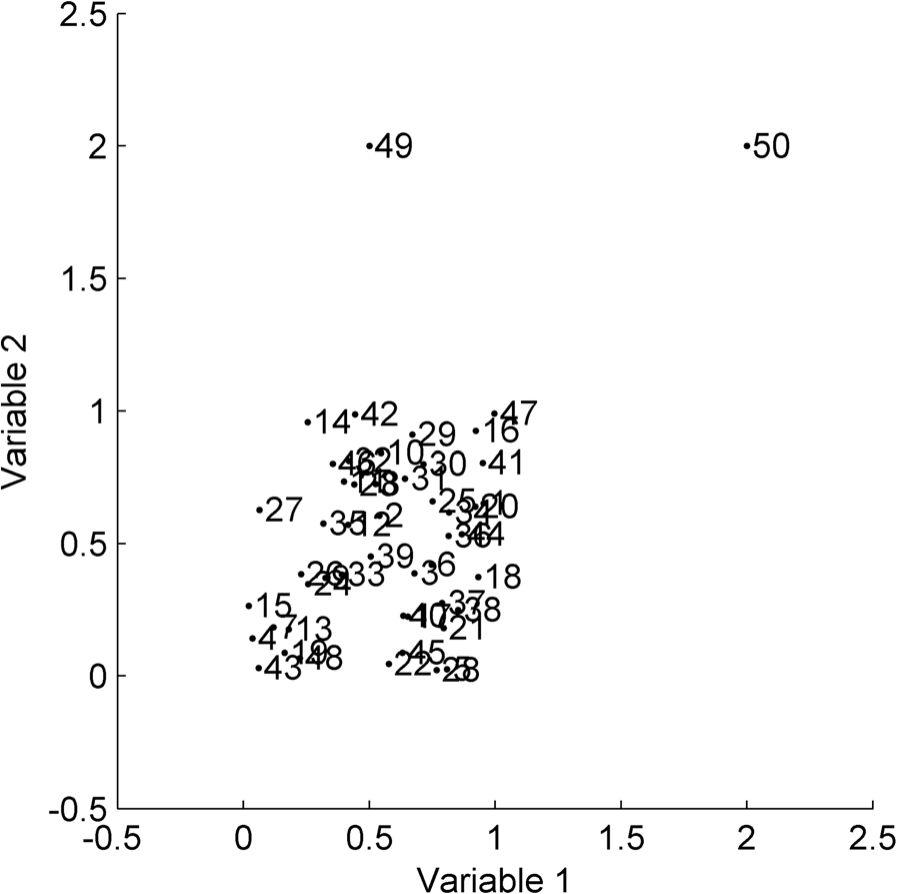
Scatter plot of the simulated dataset.

To allow a better interpretation of the proposed approach, results on a two-dimensional simulated dataset will be considered throughout the major part of this discussion and wherever applicable. As shown in Figure 1, this dataset has a cluster of 48 training samples and the remaining two training samples (49 and 50) are located quite in the extremities of the space with respect to these clustered samples.

### Defining thresholds for training samples

Thresholds have a great influence in characterising the AD for reliable predictions; a test sample that exceeds the threshold condition is associated with an unreliable prediction.

Like the adaptive kernel methods, instead of defining a general unique threshold as seen with several classical AD approaches, the proposed approach allocates a set of thresholds corresponding to the various training samples.

For a given value of *k*, threshold allocation process can be summarised as follows:

a) First of all, the distances of each training sample from the remaining *n* – 1 samples are calculated and ranked in increasing order, *n* being the total number of training samples. This will result in a *n* x (*n −*1) neighbour table **D**; an entry *D*_*ij*_ of the table corresponds to the distance of the *i-*th sample from its *j-*th nearest neighbour:

$$D_{i1}\leq D_{i2}\leq \ldots \leq D_{i,n-1}$$

b) The average distance of each *i*-th sample from its *k* nearest neighbours is calculated considering the first *k* entries in *i*-th row of the neighbour table:

(1) $$\begin{array}{l} {\bar{d}}_{i}(k)=\frac{\sum_{j=1}^{k}D_{\mathrm{ij}}}{k}\\ \;\mathrm{where},1\leq k\leq n-1\;\mathrm{and}\;{\bar{d}}_{i}(k)\leq {\bar{d}}_{i}(k+1) \end{array}$$

A vector $\bar{d}\left(k\right)$ of average distance values is then derived considering all the samples in the training set.

c) Next, a reference value (from now on referred as *Ref Val*), $\tilde{d}\left(k\right)$ is determined as follows:

(2) $$\begin{array}{l} \tilde{d}(k)=Q3(\bar{d}(k))\\ \;+1.5[Q3(\bar{d}(k))-Q1(\bar{d}(k))] \end{array}$$

where, $Q1\left(\bar{d}\left(k\right)\right)$ and $Q3\left(\bar{d}\left(k\right)\right)$ are the values corresponding to the 25th and 75th percentiles in the vector $\bar{d}\left(k\right)$, respectively [22].

d) Next, the ordered distances of each *i-*th training sample from all other *n* - 1 training samples are compared with the *Ref Val*. If the distance value of the *i-*th sample from its given *j-*th training neighbour (where 1 ≤ *j* ≤ *n*–1) is less than or equal to the *Ref Val*, then that distance value is retained, otherwise is discarded. The number *K*_*i*_ of neighbours satisfying this condition, minimum zero and maximum being *n –* 1, defines the density of the *i*-th sample neighbourhood:

(3) $$K_{i}:\;\left\{D_{\mathrm{ij}}\leq \tilde{d}\left(k\right)\right\}\;\forall j:1,n-1$$

e) Finally, each *i*-th training sample is associated with a threshold *t*_*i*_ which defines the width of its neighbourhood as:

(4) $$t_{i}=\frac{\sum_{j=1}^{K_{i}}D_{\mathrm{ij}}}{K_{i}}$$

If no distance value was retained for a given *i-*th training sample (*K*_*i*_ = 0), then its threshold *t*_*i*_ would be theoretically settled to 0, but a pragmatic solution is to set it equal to the smallest threshold of the training set.

**Figure 2:**
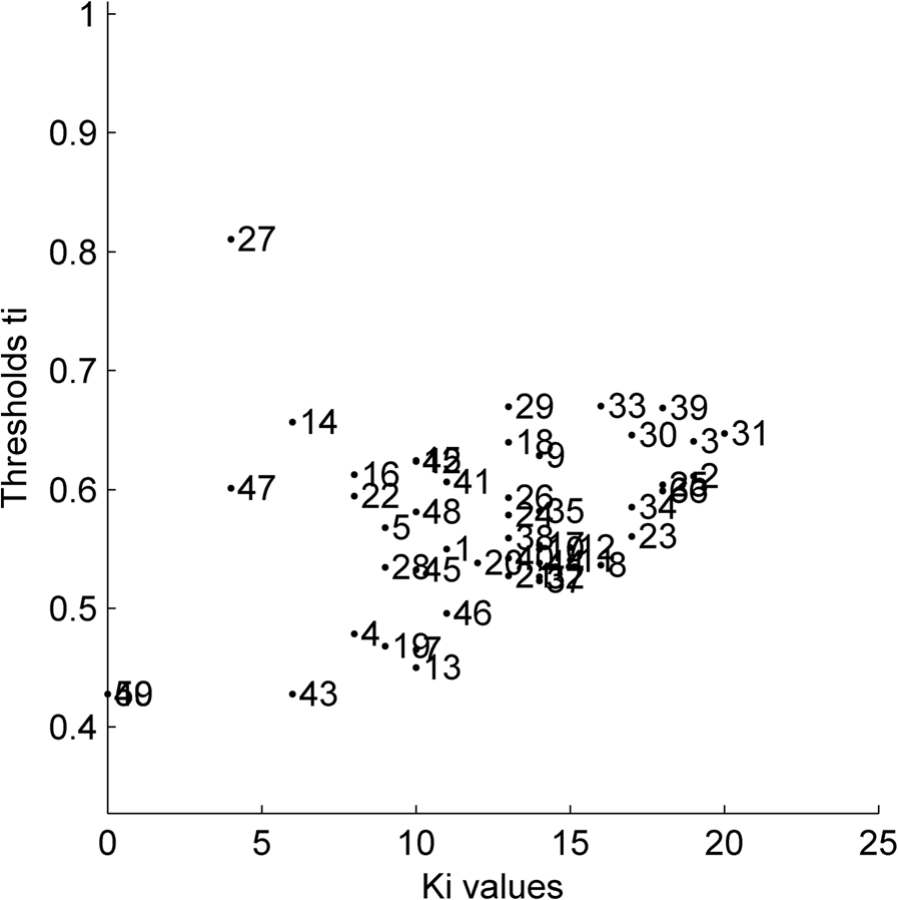
**Simulated data set.** Thresholds *t*_*i*_ vs. number of training neighbours *K*_*i*_ plot (*k* = 12).

The plot in Figure 2 provides with an overview of the thresholds for all the 50 samples in the simulated dataset. As expected, most of the training samples within the cluster (for instance, samples 2, 33 and 39) were associated with higher *K*_*i*_ values. On the other hand, obvious potential outliers (samples 49 and 50) had their thresholds equal to 0 since they couldn’t satisfy the threshold criterion even for a single training neighbour (i.e. *K*_*i*_ = 0), thus no distance values contributed to their threshold calculation. Nevertheless, they were associated with the minimum threshold equal to 0.42, i.e. the threshold of sample 43.

### Evaluating AD for new/test samples

Until this point, each training sample was associated with its individual threshold. The next step will be to characterise the AD which usually relies upon a set of conditions that will decide if a given test sample can be associated with a reliable prediction or not.

The criterion used by this approach to associate a given test sample to be within the domain of applicability can be summarised below.

Given a test sample, its distance from all the *n* training samples is calculated and simultaneously, compared to be less than or equal to the thresholds associated with those training samples. If this condition holds true with at least one training sample, the test sample will be considered inside the domain of applicability for that model. Otherwise, the prediction for that test sample will be rendered unreliable.

More formally, given the training set *TR*, for each test sample *j*, the AD decision rule is:

(5) $$j\in \mathrm{AD}\;\mathrm{iff}\;\exists i\in \mathrm{TR}:\;D_{\mathrm{ij}}\leq t_{i}$$

where *D*_*ij*_ is the distance between the *j*-th test sample and the *i*-th training sample and *t*_*i*_ is the individual threshold of the latter. In addition, each test/new sample will be associated with the number *K*_*j*_ of nearest training neighbours for which the previous condition holds true. This number can be assumed as a measure of prediction reliability; indeed, high values of *K*_*j*_ indicate that the new sample falls within a dense training region of the model’s space, while low values of *K*_*j*_ denote that the new sample still belongs to the model’s space, but located in sparse training regions. *K*_*j*_ equal to zero rejects the sample as it being outside the model’s AD since no training neighbours are identified.

**Figure 3:**
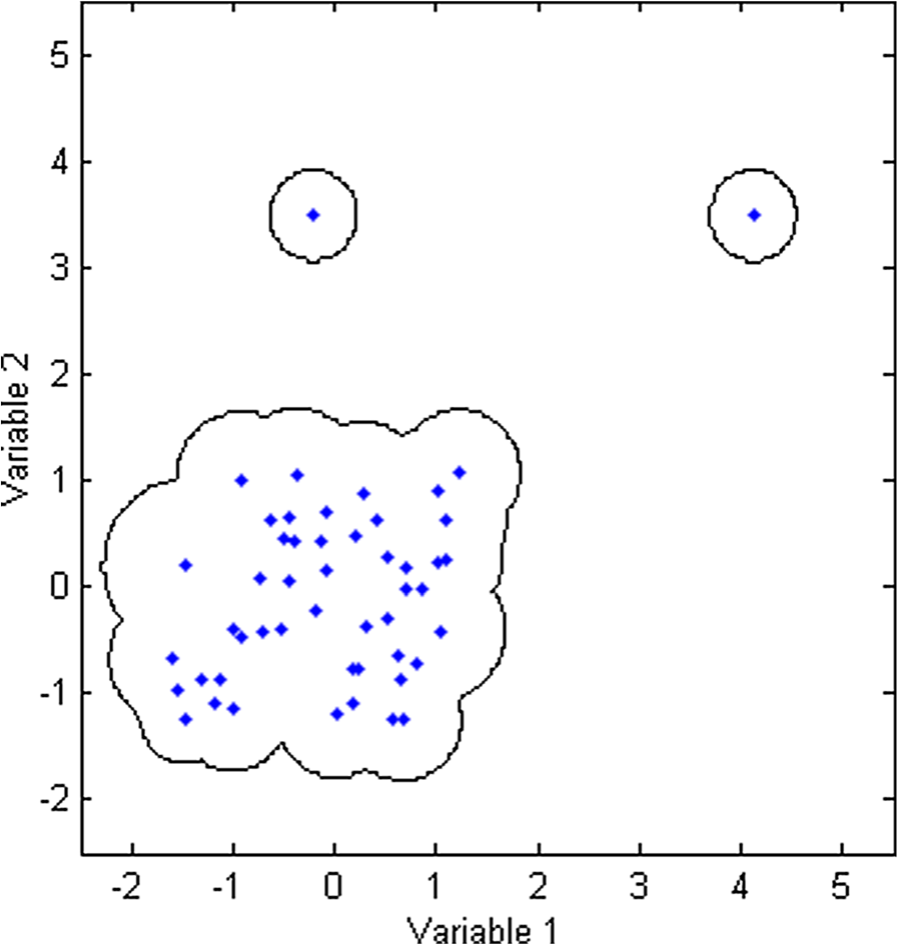
**Simulated data set.** Contour plot to demonstrate how the AD was characterised. Metric used: Euclidean distance; *k* = 12.

Figure 3 provides with the contour plot for the simulated dataset derived projecting several data points enough to fill the training space. Thresholds were calculated using 12 nearest neighbours and Euclidean distance. This choice of *k* = 12 nearest neighbours was based on the results derived performing an internal *k*-optimization, discussed later in this article. The space enclosed around the cluster represented as black line indicates that all the data points within this enclosed region were inside the AD. Thus, this region reflects in a way how the AD was characterised for this two-dimensional dataset. Area of this enclosed region tends to expand or shrink depending upon the number of nearest neighbours used for threshold calculation.

As explained earlier, the extreme outliers in the training space will be associated with the number *K*_*i*_ of neighbours equal to zero and the lowest possible threshold in the training set. Consider the sample 49 from the simulated dataset which is an extreme outlier with its threshold equal to 0.42. If there is a test sample that seems to be quite in the vicinity of this potential outlier within the descriptor space, the test sample will be associated with an unreliable prediction since its distance from sample 49 will likely exceed the small threshold. Now, consider a case, where the descriptor values for another test sample exactly overlap or are very similar to those for this potential outlier. In this situation, the distance of that sample from the outlier will be less than the threshold and thus it will be considered within the domain of applicability. In theory, this is not wrong because the potential outlier is still a part of the training space. Practically, the approach retains all the training samples to characterize the AD but minimizing the role of potential outliers in doing so. That’s the reason why the first test sample was excluded from being reliably predicted while the second sample was not. However, for the latter the number *K*_*j*_ of nearest training neighbours will likely be equal to one indicating that its prediction has some degree of uncertainty. In conclusion, there exists a relation between the defined AD and the impact of training samples in characterising it based on their threshold values.

### Optimizing the smoothing parameter *k*

Another important aspect is concerning the choice of an appropriate smoothing parameter *k*, whose theoretical range is between 1 and *n*-1.

Very low *k* values will restrict the domain of applicability in a very strict manner as compared to the AD derived opting for larger *k* values. This is because, an opted *k* value will have a direct impact on the threshold calculations which in turn can make it more rigid or easier for test samples to satisfy the threshold criterion. The strategy implemented in this article to select an appropriate *k* value was performed by Monte Carlo validation, maximizing the percentage of the test samples considered within the AD, i.e. satisfying AD criterion (Equation 5).

**Figure 4:**
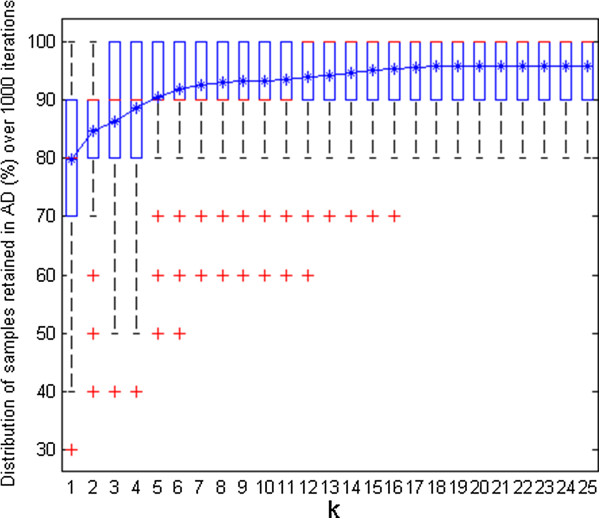
**Simulated data set.** Box-and-whisker plot of test samples (%) retained within the AD for different *k* values during *k*-optimization.

Box-and-whisker plots (box plots) were produced to get an overview of all these derived results. For instance, consider the plot in Figure 4 derived for the simulated dataset showing percentage of test samples retained within the AD with different *k* values (optimization carried out with 20% of samples in the test set and 1000 iterations).

Median quartile in the middle of the box (marked in red) can be referred for all the *k* values to get a hint about how many test samples were retained on average during the optimization process for a given *k* value. The top and bottom edges of each box plot (quartiles *Q*3 and *Q*1) correspond to 75th and 25th percentile, respectively. The whisker can extend further from *Q*1–*w*(*Q*3 − *Q*1) until *Q*3 + *w*(*Q*3 − *Q*1), of 1.5 [23]. The test samples falling outside this coverage are considered as outliers and are highlighted as ‘+’ in red. About their usefulness in the proposed AD approach, box plots showing limited spread and allowing majority of test samples to be retained within the AD can be favoured and their corresponding range of *k* values can be considered to finally opt for the most appropriate *k*. Additionally, a line plot is integrated in the same figure indicating the mean percentage of test samples that were considered within the AD for each *k* value*.* A simultaneous interpretation of both these plots can make it easier for a user to decide upon an appropriate *k* value.

Figure 4 shows that the spread of the box plots for initial *k* values is quite large. This may have resulted due to the impact of restricted training thresholds that excluded several test samples from the AD. With an increase in *k* values, the spread narrowed, however the outliers were still present until *k* = 17. After this point, the box plots remained unchanged throughout the plot with no outliers. Similar observations were derived from the mean line plot which showed a significant rise initially followed by a stable curve until the first half of the *k* values. The plot didn’t show any major changes for the second half of the *k* values. In order to avoid very high *k* values good enough to unnecessarily expand the defined AD, a *k* value of 12 was opted as appropriate *k* for this dataset. The plots dealt earlier (Figures 2 and 3) for this dataset were thus derived using this opted *k* value.

We also performed an extended analysis on several diverse data sets (results not reported in this paper), to study the influence of the smoothing parameter *k* on model’s AD definition. It was concluded that optimization of *k* can be a time-demanding procedure especially in the case of a huge number of samples, but it was also observed that this approach is quite insensitive to the smoothing parameter *k*, except for very small *k* values which led to the results influenced by local noise. Therefore, for many applications the optimization of the smoothing parameter can be avoided and reasonable results can instead be obtained by a fixed *k* value empirically calculated as *n*^1/3^.

### Reflecting the reliability in derived results

After the AD approach has been applied to the model of interest, several features will be taken into account to reflect upon the derived results. Moreover, as stated earlier the response domain will be taken into account to address the reliability in the results derived by characterising the AD of a model in its descriptor space.

In order to reflect upon a model’s predictive ability, the predictive squared correlation coefficient (*Q*^2^) was used. Since the test samples excluded from the model’s AD are unreliably predicted, in theory they should not be accounted for to calculate the model’s statistics (*Q*^2^).

The following key parameters were evaluated:

a) Number of test samples retained within the AD.

b) *Q*^2^ calculated from the test samples retained within the AD [24,25]:

(6) $$Q^{2}=1-\frac{\left[\sum_{j=1}^{n_{\mathrm{TS}}}{\left({\hat{y}}_{j}-y_{j}\right)}^{2}\right]/n_{\mathrm{TS}}}{\left[\sum_{i=1}^{n_{\mathrm{TR}}}{\left(y_{i}-{\bar{y}}_{\mathrm{TR}}\right)}^{2}\right]/n_{\mathrm{TR}}}$$

where, *y*_*j*_ is the measured response value for the *j*-th sample and *ŷ*_*j*_ its predicted value; *n*_TR_ and *n*_TS_ represent the total number of training and test samples, respectively, and ${\bar{y}}_{\mathrm{TR}}$ is the mean response of the training set.

c) List of all the test samples considered outside the AD.

d) For each *j*-th test sample, the absolute standardized error calculated as:

(7) $$SE_{j}=\frac{\left|y_{j}-{\hat{y}}_{j}\right|}{s_{Y}}$$

where, *y*_*j*_ is the measured value for the *j*-th sample and *ŷ*_*j*_ its predicted value; *s*_*Y*_ the standard error of estimate derived from the training set.

e) The information about how many times the threshold criterion (Equation 5) is satisfied by each test sample, that is, how many training neighbours (i.e. *K*_*j*_) are located at a distance less than or equal to their threshold values, from a given test sample.

In theory, a test sample satisfying the threshold criterion several times (i.e. having high *K*_*j*_) is expected to be predicted with higher accuracy. This can be desired since less distant training neighbours indicate a higher structural similarity of the test sample. On the contrary, a test sample satisfying the threshold criterion for no training neighbours (*K*_*j*_ = 0) indicates that there wasn’t any training sample similar enough to reliably predict that test sample.

## Results and discussion

As the case study to derive results with the proposed strategy, the CAESAR Model 2 to predict bioconcentration factor (BCF), which was developed under the EU project CAESAR following the REACH requirements, was selected. It is a Radial Basis Function Neural Network (RBFNN) model derived from 378 training and 95 test samples [20,21]. The five descriptors used to develop this model were calculated using Dragon 5.5 [26].

The statistics for this model are summarized in Table 1.

**Table 1:** Summary of model statistics for the case study

***Model***	***Training set***			***Test set***		
	***n***_***TR***_	***R***^***2***^	***RMSE***	***n***_***TS***_	***Q***^***2***^	***RMSEP***
CAESAR Model 2	378	0.804	0.591	95	0.797	0.600

*R*^*2*^ Determination coefficient; *RMSE* Root-mean-square error; *Q*^*2*^ Predictive squared correlation coefficient; and *RMSEP* Root-mean-square error of prediction.

For comparison purposes, some AD approaches taken from literature [2,7-10] were implemented on the selected case study. Among them, the classical kNN-based AD approach [6,18] was implemented by calculating average distances of all the training samples from their 5 nearest neighbours (i.e. *k* = 5); since the choice of thresholds didn’t follow any strict rules in the existing literature, the value corresponding to 95th percentile in this vector of average distances was considered as general threshold. If the average distance of a test sample was lesser than or equal to the threshold value, the test sample was retained within the AD.

In addition to the classical kNN-based AD approach, the following methodologies were considered [2,7-10]: the Bounding Box, which is based on the ranges of model variables; its variant based on principal components instead of the original variables (PCA-Bounding Box); the Convex Hull, which is the smallest convex area that contains the original set; two distance-based methods, which calculate the distance (Euclidean and Mahalanobis) of a test sample from the data centroid and use the 95th percentile of the training sample distances as threshold.

Finally, some methods for probability density function estimation were also considered. Among the multivariate kernel density methods, four variants of Gaussian kernel estimators were implemented [19]: fixed Gaussian kernel with bandwidth equal to 0.462 (for the studied data set); optimized Gaussian kernel with a smoothing parameter equal to 0.237 obtained by leave-one-out cross-validation [27]; variable Gaussian kernel with bandwidth calculated as the inverse function of the Euclidean distance to *k*-th neighbour (*k* = 15) [27]; adaptive Gaussian kernel, with fixed Gaussian kernel as the pilot estimate and sensitivity parameter α equal to 0.5. Finally, Epanechnikov kernel with a fixed bandwidth equal to 1.961 and the nearest neighbour density estimator with smoothing parameter *k* equal to 15, were also considered [19].

For all the implemented methods, except for Bounding Box and Convex Hull, autoscaling was adopted as data pretreatment.

**Figure 5:**
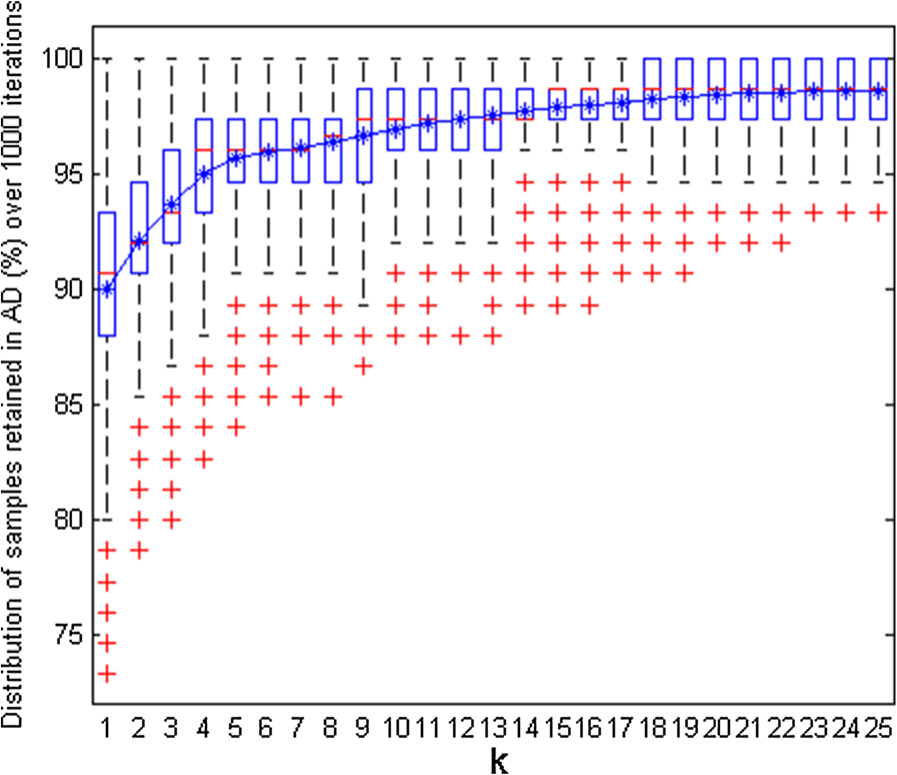
**CAESAR BCF model.** Box-and-whisker plot of test samples (%) retained within the AD for different *k* values during *k*-optimization.

The proposed AD strategy was implemented in MATLAB [28] using autoscaled Euclidean distances. The *k*-optimization procedure was carried out initially to decide upon an optimal *k* value; the training set of 378 samples was randomly partitioned 1000 times selecting 20% of samples in the test set (i.e. 75 samples). The box plots in Figure 5 summarize the percentage of test samples retained within the AD for different *k* values (up to 25).

As expected, the first lower *k* values were associated with box plots having highest spread. This degree of dispersion lowered gradually with increase in number of neighbours considered. The line plot of the mean showed an increase in the number of samples throughout the plot, however, this increment after initial *k* values was gradual. Based on their lower spread and preference to retain reasonably higher number of samples within the AD (as reflected from their median), the *k* values in the range of 15–19 were considered further to decide upon an optimal *k*. Finally, to avoid unnecessarily higher training thresholds and their resulting impact on the defined AD, *k* = 15 was considered as an optimal choice for this case study. Considering the selected *k* value of 15, the novel AD approach identified four test samples (33, 61, 82 and 83) being outside the model’s AD.

**Figure 6:**
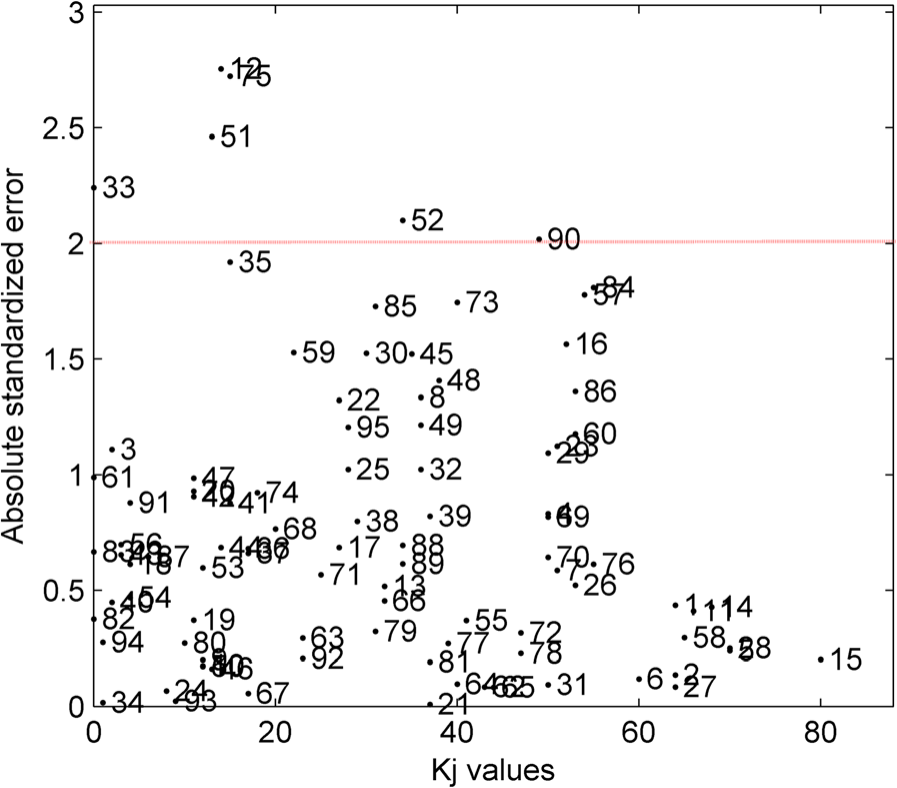
**CAESAR BCF model.** Absolute standardized error of test samples plotted against their *K*_*j*_ values.

To reflect upon the reliability in the results derived with this approach, absolute standardized error of all the test samples was plotted against their corresponding *K*_*j*_ values. As shown in Figure 6, four test samples considered outside the AD with this approach were associated with a value of *K*_*j*_ = 0. The absolute standardized error for sample 33 was quite higher as compared to the remaining three samples. As seen clearly from the plot, there had been a sharp decrease in the prediction error of test samples with an increase in *K*_*j*_. However, it can’t be denied that this pattern wasn’t rigidly followed in the results. There had been test samples with very low *K*_*j*_ values but extremely low or negligible absolute standardized error, meaning that even less reliable predictions can have good accuracy. In any case, this plot somehow tried to interpret the AD results derived in model’s descriptor space taking into account the response domain, and clearly informed about both, reliability and accuracy in the predictions of the test samples. Most of the predictions had good accuracy, them being within two units of standardized error and high reliability. The samples corresponding to these reliable predictions were associated with higher *K*_*j*_ values, thus being well represented by several training samples.

Generally, a standardized error of two/three units is usually considered as a warning value for outliers detection. In Figure 6, six test samples (12, 33, 51, 52, 75 and 90) exceeded a two-unit threshold for the absolute standardized error indicating them as outliers in the model’s response domain. It can be interesting to further evaluate the reasons behind categorising them as outliers; however, this is beyond the scope of this article as the proposed AD approach is defined within the model’s descriptor space. Nevertheless, this evaluation identifies sample 33 as an outlier in model’s descriptor’s space as well as its response domain which further supports the results derived from the proposed approach to exclude this sample from the model’s AD.

Finally, the results derived by this approach were compared with those derived from classical AD approaches. Table 2 reports these results; the first row shows results when no AD approach has been applied to bound the model’s descriptor space.

**Table 2:** Comparison of AD methods applied to the test set of CAESAR BCF model

***Approach***	***IN AD***	***Q***^***2***^	***OUTSIDE AD***
All samples inside (no AD approach)	95	0.797	None
Proposed approach (Euclidean dist., *k* = 15)	91	0.803	33 61 82 83
Bounding box	95	0.797	None
PCA bounding box	93	0.804	33 40
Convex hull	73	0.789	3 7 9 13 18 33 34 36 37 38 39 40 41 43 51 56 61 72 79 91 92 94
Euclidean dist (95 percentile)	88	0.802	3 33 36 37 40 42 61
Mahalanobis dist (95 percentile)	89	0.791	18 43 54 61 83 91
Classical kNN (Euclidean dist., *k* = 5)	87	0.797	3 33 34 40 61 82 83 94
Fixed Gaussian kernel	85	0.794	3 24 33 34 40 61 82 83 91 94
Optimized Gaussian kernel	66	0.831	3 912 22 24 33 34 38 40 45 47 51 53 54 56 61 68 69 75 76 80 82 83 87 89 91 93 94 95
Variable Gaussian kernel (*k* = 15)	81	0.790	3 24 33 34 40 43 61 80 82 83 89 91 94 95
Adaptive Gaussian kernel	88	0.801	3 33 43 61 82 83 91
Fixed Epanechnikov kernel	87	0.799	3 33 40 43 61 83 91 94
Nearest neighbour density estimator (*k* = 15)	91	0.806	3 33 61 91

The number *k* of nearest neighbours considered with the proposed approach (i.e. 15) was comparatively higher than the one considered with classical kNN (i.e. 5); however, the impact on model statistics was not so obvious on the resulting *Q*^2^, while the number of retained samples increased from 87 (classical kNN) to 91 (proposed approach). Discussing the results derived with classical approaches, number of samples retained within the AD varied significantly depending on what strategy was used. Convex hull, optimized and variable Gaussian kernel methods retained the least number of samples while the Bounding Box considered none of the test samples outside the AD. Overall, the proposed approach worked quite well on the CAESAR model, trying to define an AD with maximum retained test samples within the domain and positive impact on the model statistics.

The last column of Table 2 reports the list of samples considered outside the AD with all the approaches. Irrespective of total number of samples considered outside the AD, all the methods converged significantly identifying a subset of common samples that were always excluded from the model’s AD.

## Conclusions

A novel kNN-based approach to define the AD of QSAR models was proposed. The overall execution of this approach was performed in three different phases that efficiently used the salient features of kNN principle to define a model’s AD in its descriptor space. Significant features that distinguished the proposed AD approach include defining individual threshold for each training sample, optimizing the smoothing parameter *k* to be considered and taking into account the model’s response domain to reflect upon the reliability of results derived in its descriptor space.

In the proposed AD method, the appropriate number *k* of neighbours can be chosen on the basis of the plot with retained samples vs. *k* values obtained by Monte Carlo validation; it allowed to identify a smoothed region of the *k* values where the results remained unchanged, ensuring high robustness in the AD definition.

The results on the selected case study defined an AD with a positive impact on model statistics retaining maximum possible samples that were reliably predicted. Comparison of the derived results with those from the classical approaches by no means intended to project the pitfalls of existing approaches but it was aimed to have a performance evaluation of this novel strategy to understand how its implementation could lead to obtain similar or different results as compared to the classical ways of defining the AD. An extended comparison of the different AD approaches on several diverse data sets have indicated the following relevant features that characterize the proposed AD approach: a) adaptability to local density of samples, useful when the underlying multivariate distribution is asymmetric, with wide regions of low data density; b) unlike several kernel density estimators, effectiveness also in high-dimensional spaces; d) low sensitivity to the smothing parameter *k*; d) versatility to implement various distances measures other than Euclidean distance, such as Manhattan distance, Mahalanobis distance and the recently proposed locally-centred Mahalanobis distance [29], depending on the data set in analysis.

A MATLAB module for the model’s AD estimate by different approaches will be soon available at http://michem.disat.unimib.it/chm/.

## Competing interests

The authors declare that they have no competing interests.
